# The Talent Training Mode of International Service Design Using a Human–Computer Interaction Intelligent Service Robot From the Perspective of Cognitive Psychology

**DOI:** 10.3389/fpsyg.2021.600218

**Published:** 2021-02-02

**Authors:** Yayun Yang

**Affiliations:** School of Architecture, Taiyuan University of Technology, Shanxi, China

**Keywords:** artificial intelligence, robot, talent training mode, cognitive psychology, talent training

## Abstract

To effectively improve the efficiency of international service design talent training and make it more in line with society's needs, we analyze the current status of international service design talent training and its professional training focus. Based on the above problems, from the perspective of cognitive psychology, artificial intelligence and human–computer interaction (HCI) technology are used to construct the international service design talent training mode of the HCI intelligent service robot. This mode can be used to solve the existing teaching problems by using novel means to ensure the quality of teaching. Finally, through the actual analysis of teaching cases, the effectiveness of the proposed talent training mode is verified. The HCI system is based on knowledge of cognitive psychology. According to the characteristics and functions of an educational robot, the robot is combined with traditional teaching activities, and the robot-assisted talent training mode is designed. Robot-assisted talent training is a feasible training method that can improve the efficiency of talent training. Students have confidence in their learning skills before the course, and the confidence is further strengthened after the end of the course. After the course, the students have a stronger sense of cooperation. This study can provide theoretical ideas for the research of international service talent training mode.

## Introduction

The deep integration of information technology and the manufacturing industry will inevitably give birth to a group technological revolution with intelligent manufacturing as the core and new energy and new materials infiltrating each other. A new production mode, industrial form, and business model will also form (Wang and Han, 2020). The rapid implementation of digitization, networking, intelligence, and service will certainly improve the product design ability and quality and promote the rapid transformation of production mode and industrial form in various fields (Qi and Tao, 2018). Developed countries have carried out rapid planning and distribution in industries that are conducive to their own development to adapt to leading a new era of scientific, technological, and industrial revolution (Sani et al., 2020). In particular, for the development of science, technology, the economy, and higher education institutions of talent training, a new professional talent training mode is proposed (Xu et al., 2019). Therefore, the study of the talent training model is of great significance to ensure the sustainable development of the economy and society.

International service design majors should pay attention to stakeholders, products, and services from the perspective of service to meet the needs of all stakeholders and achieve a good state of sustainable development (Pacheco et al., 2019). International service design has gradually become the focus of academic and industrial circles (Talevski et al., 2018). However, at present, the knowledge system and ability system of the specialty and talent training need to be adjusted and updated. In terms of talent training objectives, talent training programs, curriculums, and teaching evaluations, it is not only necessary to emphasize the basic, personalized, practical, integrated, and service-oriented cultivation but also to strengthen the guidance of students' innovation consciousness, social responsibility, industry-university-research and multi-dimensional professional evaluation (Grenha Teixeira et al., 2017), which can better cope with the new needs of international service design talent.

With the advent of the information age, the application of information technology in the field of education has gradually penetrated various disciplines. The combination of artificial intelligence (AI) and robot technology will bring breakthroughs to the robot industry (Bin and Mandal, 2019). Kuo et al. (2017) used AI robots in hotel services and found that hotel industry in Taiwan has good potential to implement service robots. A robot service can help hotels deal with seasonal employment and labor utilization (Kuo et al., 2017). Luo et al. (2018) analyzed the influence of AI on the accounting industry's development and put forward relevant suggestions for the problems existing in AI (Luo et al., 2018). Yu et al. (2019) proposed active design research based on an AI technology development platform. The platform can help students to develop learning plans and register for required courses (Yu et al., 2019). Liu and Wang (2020) established an AI education informatization teaching model, read and collated a large number of literature, such as big data and ice snow talent training, constructed a visual analysis framework of AI teaching data, and discussed the realization process and mechanism of data visualization of ice and snow mixed talent training from two dimensions (timeliness and media form) (Liu and Wang, 2020). Pillai and Sivathanu (2020) proposed a model to explore the use of Adobe Illustrator technology for talent training. Research shows that the use of AI technology in talent training has a positive impact, which can effectively reduce costs and effectively improve teaching efficiency (Pillai and Sivathanu, 2020). It shows that AI technology has been applied to talent training in many studies, and good experimental results have been achieved.

Therefore, in order to change the existing teaching methods of international service design talents and effectively improve the efficiency of teaching and talent training, the relevant theories are analyzed on the basis of previous studies, and the feasibility of the application of AI and robots to the international service design talents training mode is explained from the perspective of cognitive psychology. The questionnaire is used to verify the application effect of robots in the international service design talent training mode. This study can provide a theoretical basis for international service design talent training.

## Method

### Current Situation of International Service Design Talent Training

**Figure 1:**
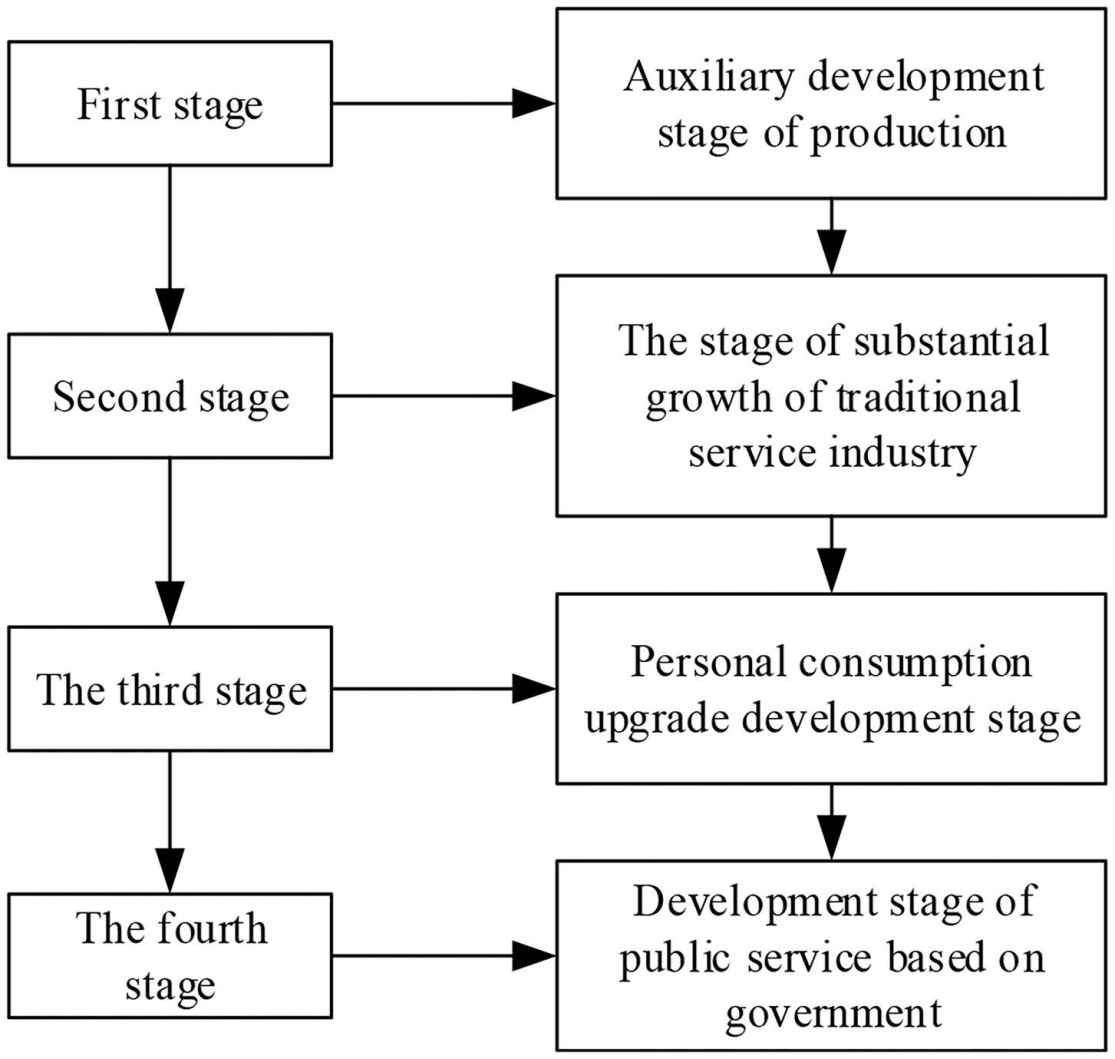
Four stages of service-oriented economy development.

The international service design talent training mode in domestic colleges and universities is still in the pipeline. University education is a highly specialized semi-closed teaching method. On the one hand, teaching is open to factories and enterprises, emphasizing practical teaching and teaching practice. On the other hand, the self-closing and automation mode of different discipline systems in schools begins to emphasize general education on the basis of professional education. The university's teaching of different departments and disciplines begins to move toward limited opening, forming a new era of semi-open talent training (Yu and Sangiorgi, 2018). With the development and change of economy, the content of service design is also different. The development of a service-oriented economy has experienced the following stages, as shown in Figure 1.

The core of service design is to provide value for users, and products and interaction are essential ways to realize service value (Touloum et al., 2017). Service design can be divided into three core values: caring for people, responding to unforeseen needs, and providing access rights for people. The three core values of service have the characteristics of overlapping and mutual influence, and the process of service design is nonlinear and iterative (Iriarte et al., 2018). Generally speaking, professional service design includes the scenario study stage, service innovation stage, and organization implementation stage. These three stages are crucial, each with their own workload and expertise. Among them, interdisciplinary collaboration runs through the whole service process.

The teaching of international service design courses and teachers' knowledge structure in domestic colleges and universities are seriously lagging. At present, many international service design teachers adopt the traditional teaching model, so teachers themselves have the ideology and tendency of emphasizing external form and neglecting internal ideological root (Kohlbry, 2016). This requires that, in the formulation and design of professional talent training objectives, curriculum, classroom teaching management, and guidance, teachers have a wide range of knowledge, rich information content, and an improved knowledge structure rather than being limited to the scope of their professional research. Teachers lack integration with related disciplines and also inevitably lack the spirit of innovation (Chydenius, 2020). It is very difficult for backward teachers to cultivate compound talents with high professional quality and strong adaptability; adapting to the industrial era will inevitably bring great resistance to the development of the major and the cultivation of talents, affecting the teaching of professional courses and the employment of students.

### Analysis of Cognitive Psychology

**Figure 2:**
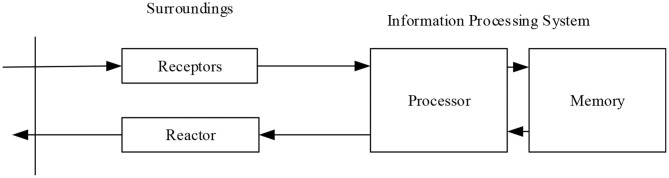
Structural model of cognitive psychology.

Cognitive psychology is a psychological science that studies the mental processing behind cognition and behavior (including thinking, decision, reasoning, and the degree of motivation and emotion). This science covers a wide range of research fields, aiming to study the operation of memory, attention, perception, knowledge representation, reasoning, creativity, and problem-solving (Ritter et al., 2017). According to the theory, the information-processing system of the human brain is composed of four parts: receptor, effector, memory, and processor (or control system). First, the environment inputs information into the receptor, and the receptor converts the information. Before entering the long-term memory, the converted information needs to be symbol reconstructed, identified, and compared by the control system. The memory system then stores the symbol structure that can be extracted. Finally, the effector reacts to the outside world (Zwaan et al., 2018) it was shown in Figure 2.

The field of education uses service design. The receivers of teaching services are students, and they are thus the main target users of service design. The purpose is to tap students' potential needs to improve the existing teaching experience and quality and meet the teaching requirements. This exploration focuses on the design of a student-centered teaching service. The characteristic of student-centered teaching service design is to let students directly participate in the design. The student-centered teaching design method aims to integrate the needs of students into the development process of teaching service design, which is a supplement to the existing teaching plans and means. In the theory of service design, service-oriented technologies, such as Internet grid technology, service technology, and cloud computing, are in the process of development and change. However, the whole design process should closely focus on users and attach importance to the user experience to improve user satisfaction and increase the popularity of service design (Greenberg et al., 2017). Cheng et al. (2019) designed a talent training model for cross-border e-commerce by combining problem-based learning and the social media cognitive process. They developed a procedure to evaluate the effectiveness of the model. The actual verification proves that, in the service design, the cognitive psychology of users can be used to effectively improve students' perception and attention (Cheng et al., 2019).

In service design, students' cognitive psychology can be used to transform students' perception, attention, memory, reaction, and other requirements into design points, which can be introduced into the process of service design. It can enhance students' learning ability, improve service level, and facilitate product updates and iteration. The design of the human–computer interface will affect the user's sense of use and satisfaction in the operation process. In the process of human cognition, the stimulus information transmitted by the interface is received through a variety of sensory channels. Then, through the attention mechanism, the received information is effectively selected, and the conversion from instantaneous storage to long-term storage is completed. Finally, the information is encoded and stored by the storage system (Van Doorn et al., 2017).

### AI Analysis

AI includes psychology, philosophy, neurophysiology, computer technology, and many other areas of knowledge. An AI device simulates human perception, learning, reasoning, communication, and other complex activities (Luo et al., 2019). The difference between AI and natural human intelligence is that an AI can save a lot of organic activities. An intelligent machine developed by AI technology can simulate the function of human organs and can replace simple, repetitive, and even complex work that is difficult to complete in daily life, thus changing people's social needs (Deng, 2018).

AI education is the product of the deep integration of AI and education. In educational activities, people use intelligent technology to improve teaching quality, accelerate the development of education and teaching, create a new teaching environment, provide personalized teaching services for schools, and achieve the purpose of education by realizing the student-centered teaching concept (Makridakis, 2017). The current intelligent classroom is the product of AI. In the intelligent classroom, the Internet of Things architecture, network control mode, touch control terminal, and convenient and efficient Internet technology are used to realize the interconnection between teachers' computers and students' terminals, and real-time interactive teaching is carried out.

This mode has a flexible multi-screen interaction and video display switching mechanism, which can flexibly realize the purpose of multi-device display switching and complete teaching tasks through a variety of teaching methods (Bruya and Ardelt, 2018). AI technology provides support for teaching, provides a good platform for learners, improves the teaching effect, realizes the evaluation of teachers' information resources and students' learning status, and puts forward corresponding learning suggestions for each student (Bajaj and Sharma, 2018). From the perspective of technology development, the development of AI can be divided into three stages: computational intelligence, perceptual intelligence, and cognitive intelligence. Among them, computational intelligence is the initial form of AI, and it is also the basis for its continuous development. Perceptual intelligence is the stage in which the development of AI is concentrated in China and foreign countries. Cognitive intelligence is the advanced form of AI, which is the breakthrough of future development of AI.

In the teacher's console, the advanced network system control architecture is adopted; it can realize the remote control of various equipment and allow teachers to control unexpected situations in the classroom and adjust accordingly. At present, AI technology has been widely used in teaching activities. The educational robot is one of the products of AI technology and human–computer interaction (HCI) technology that can improve the efficiency of talent training.

### Analysis of HCI

**Figure 3:**
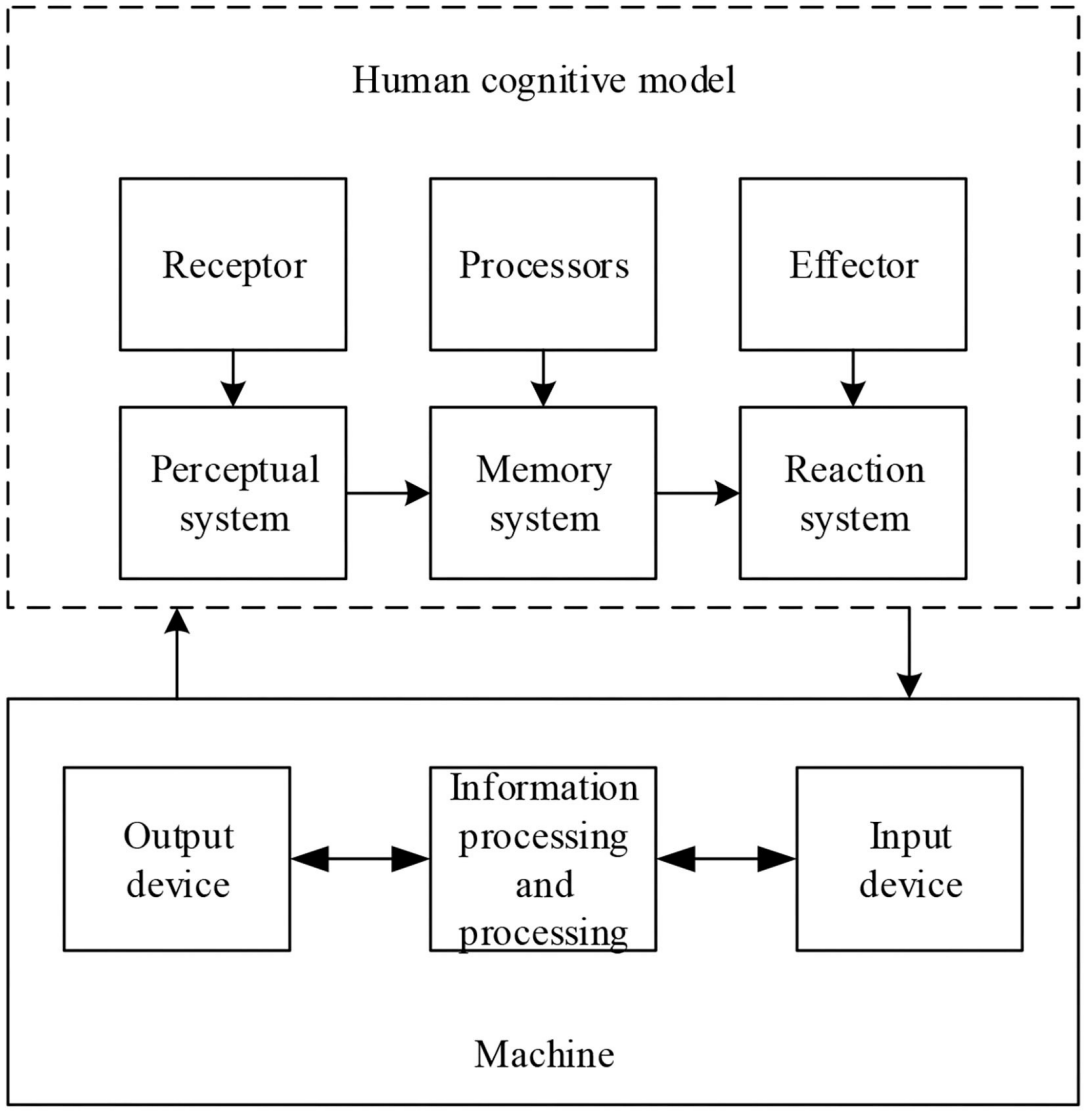
Workflow chart of HCI system.

In the HCI system, people and machines perform their respective duties and use their respective processing systems to process information. The first is the perception interface. The human perception system will receive visual, tactile, or auditory stimuli presented by the machine interface, select and process the stimuli presented by the machine interface, and transmit them to the cerebral cortex for memory. The second is the storage system. Through the human storage system, the information obtained is stored, processed and extracted, and transmitted to the human response system. Finally, HCI is implemented. Through the HCI system, the machine interface can be operated accordingly, and the received stimulus can be fed back to the human perception system (Kuo et al., 2019). Figure 3 shows the workflow of the HCI system.

HCI is characterized by repeatability, flexibility, digitization, humanoid appearance, body movement, interaction, and personification. When HCI is used in personnel training, it can carry out simple and repeated operations to help the talents get familiar with and master the knowledge of service design. It can make use of flexibility to help teachers adjust and design teaching activities according to relevant teaching requirements so that teachers and students are no longer restricted by learning content and teaching materials. In terms of digitization, it can use the sharing and preservation characteristics of digital data to record teachers' teaching experience so as to serve personnel training (Khan, 2016) better. A humanoid appearance is more attractive, which can stimulate students' curiosity and fantasy and improve their initiative; body movement can guide learning content through action; and HCI can interact with students to improve their initiative and practicability. The personification of HCI can also to an extent avoid student embarrassment during the learning process (Urquiza-Fuentes and Paredes-Velasco, 2017).

### STEM Education

STEM is the abbreviation of science, technology, engineering, and mathematics. Among them, science involves understanding the world and explaining the objective laws of nature; technology and engineering are made to transform the world, realize the control and utilization of nature, and solve the problems encountered in the process of social development on the basis of respecting the natural laws; and mathematics is the basic tool of technology and engineering. A STEM curriculum focuses on strengthening the education of students in four aspects. The first is scientific literacy, that is, the use of scientific knowledge (such as physics, chemistry, biological science, and geospatial science) to understand nature and participate in the process of influencing nature. The second is technical literacy, that is, the ability to use, manage, understand and evaluate technology. The third is engineering literacy, which is the understanding of the process of technical engineering design and development. The fourth is mathematical literacy, which is students' ability to discover, express, explain, and solve mathematical problems in a variety of situations. Among science, technology, engineering, and mathematics, there is a relationship of mutual support, supplement, and development. To understand them—especially the relationship between them—they cannot be separated. Only in their interaction and mutual collision can deep learning and understanding be realized and cultivated.

The STEM teaching method also attempts to change the typical teacher-centered classroom model and encourage the development of a problem-solving, exploration and discovery learning curriculum model. This model requires students to actively seek solutions to problems. In recent years, the number of college students majoring in science and technology and related fields has decreased significantly. On the one hand, it is due to the lack of warm-up in the high school classroom. On the other hand, in addition to classroom teaching, these majors also require the completion of high-intensity experimental courses. Due to inadequate preparation in all aspects, the students give up the major they want and invest in the courses and majors with a relatively low difficulty coefficient. In the long run, there will be a labor shortage in the field of engineering science. The four parts of STEM have been in the mode of separate teaching and are, in most cases, independent of each other. However, the STEM teaching method breaks the disciplinary boundaries of science, technology, engineering, and mathematics so that they complement each other and complete the whole teaching together. Science, engineering, and mathematics are perfect because of technology. At the same time, the technology discipline also provides an innovative way to solve problems.

### Training Mode of International Service Design Talents Based on HCI

**Figure 4:**
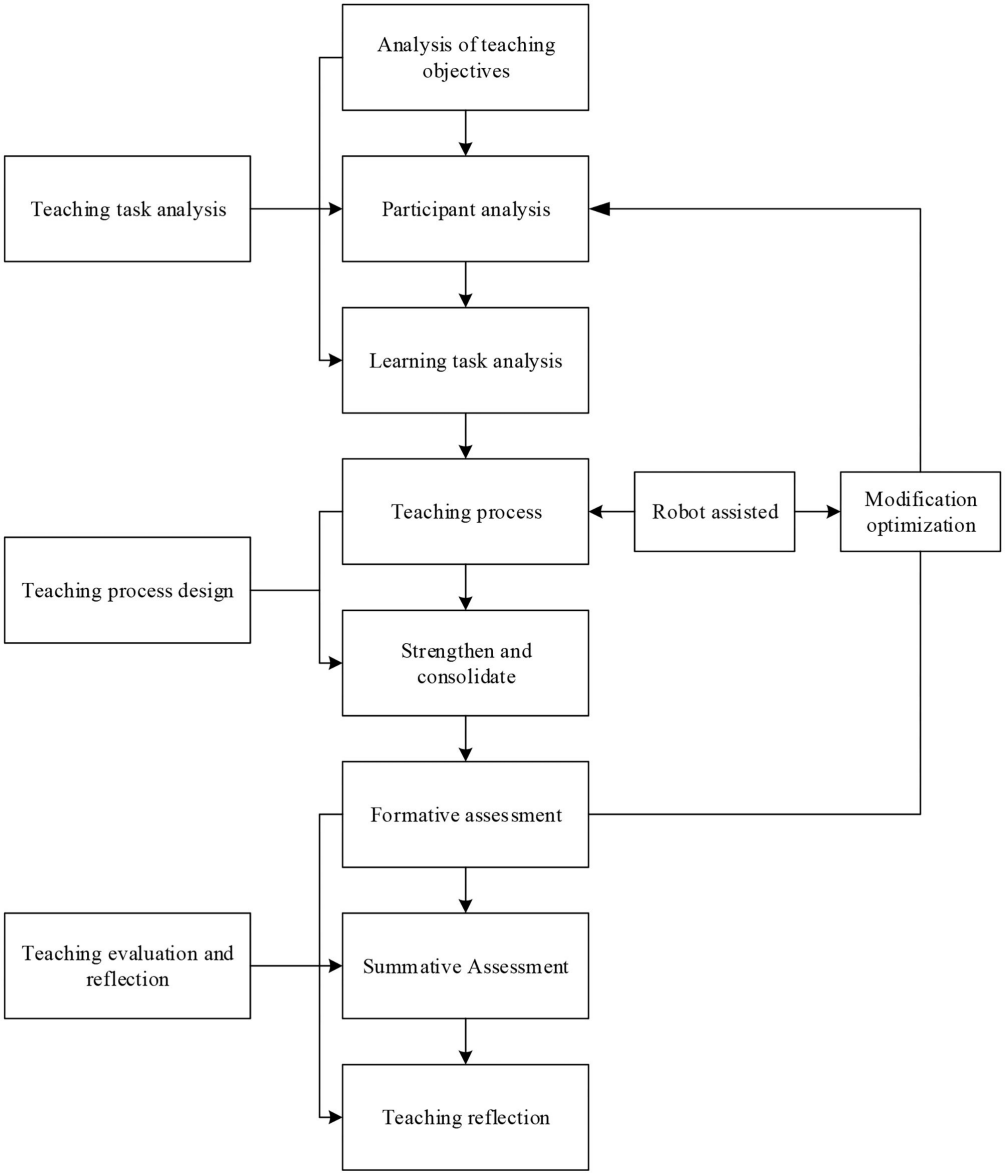
Talent training mode based on intelligent service robot.

The HCI-based international service design talent training process is divided into three parts: teaching task analysis, teaching process design, and teaching evaluation and reflection. Among them, teaching tasks analysis is the basis of the whole teaching design. The teaching tasks analysis includes the analysis of teaching objectives, the analysis of participants, and the analysis of learning tasks. The teaching process design is to improve the use of the technical advantages of robots to create a real learning situation, formulate appropriate learning strategies, and help talents to carry out independent inquiry learning by using learning resources and tools. Teaching evaluation and reflection is used to evaluate the learning effect of students and the overall situation of teaching to optimize the teaching design scheme, which is an essential link in teaching design. Figure 4 shows the talent training mode based on an intelligent service robot.

Among them, teaching task analysis includes teaching objective analysis, learning task analysis, and participant analysis. Teaching process design includes learning situation design, teaching resource design, and learning strategy design. Teaching evaluation is divided into formative evaluation and summative evaluation. Teaching reflection refers to the last step in the design of teachers' teaching activities, which is also an indispensable step.

### Teaching Case Verification

(1) Curriculum design: A total of 16 robot experiments are carried out for 36 class hours. The research is mainly based on the theme project of international service design. Due to the limitation of teaching space and experimental equipment and the large number of students (41 in total) selecting this course, the course is taught in batches; 41 students are divided into groups according to the diversified and voluntary combination rules. There are 10 groups with 3–4 people in each group and 3–4 groups in each batch with 3 batches. The teaching activities of the teaching project are designed and implemented according to the three parts of the robot teaching activity design model. The teaching site is in the teacher's laboratory, which can only accommodate 10 people each time for teaching. The experimental site and experimental equipment are limited so students are required to carry personal notebook computers, which not only solves the problem of insufficient resources but also facilitates students' learning after class. The software and equipment related to the experiment are provided or prepared by the teaching assistant after class.
(2) Data source: in this survey, students from two schools in city C are selected as the research objects. Among them, the questionnaire survey is for all students, and the semi-structured interview is to select the excellent and backward students of each grade from the two schools for interview. The two selected schools are school A and school B. Table 1 shows the basic distribution of students participating in the questionnaire survey in the two schools.

**Table 1:** Basic distribution of students participating in the questionnaire survey in two schools.

**School**	**Level 1**	**Level 2**	**Level 3**	**Total**
School A	Male 272	Male 258	Male 171	Male 701
	Female 205	Female 198	Female 206	Female 609
	Gender not written 10	Gender not written 9	Gender not written 10	Gender not written 29
Total	487	465	387	1339
Invalid questionnaire	14	21	62	97
School B	Male 73	Male 79	Male 131	Male 283
	Female 130	Female 91	Female 183	Female 404
	Gender not written 333	Gender not written 269	Gender not written 30	Gender not written 632
Total	536	439	344	1319
Invalid questionnaire	10	17	28	55
Total valid questionnaire	1,023 + 24 (Invalid questionnaire)	904 + 38 (Invalid questionnaire)	731 + 90 (Invalid questionnaire)	2658 + 152 (Invalid questionnaire)

In this exploration, 2,810 questionnaires are distributed, and 2,658 valid questionnaires and 152 invalid questionnaires are collected. In the process of answering questions, if there is an obvious careless phenomenon, it will be regarded as an invalid questionnaire. In school A, of the 12 freshmen classes enrolled in autumn, 2 classes are randomly selected as experimental classes with 55 and 56 students, and the remaining classes are the control class. In school B, among the nine freshmen classes enrolled in the autumn, two classes are randomly selected as experimental classes with 55 students in each class. The other classes are the control class.

(3) Survey method: through the open-ended interview method, the students with different academic achievements (excellent, medium, and backward) are interviewed. First, the concept of learning strategy is put forward to the students so that they can think about which behaviors are helpful to improve learning efficiency and which behaviors affect learning. Then, each student is asked to write at least five related items. Six forums are held in six schools, with about 30 participants in each forum. The students participating in the forum represent different grades of academic achievement, from excellent to backward, with about 10 students in each grade. Through interviews, 86 effective learning behaviors and 64 unfavorable learning behaviors are obtained.

These items are sorted out into a learning strategy questionnaire. The process of sorting out is to retain the projects with high recognition, eliminate the projects with low recognition (fewer than five people), and then carry out the single dimension treatment of the items, that is, an item can only represent one event content. Thus, 98 descriptive sentences about learning strategies are obtained as the measurement items of the questionnaire. The questionnaire mainly observes the difference in learners' comprehensive quality from five dimensions: learning interest, learning skills, classroom participation, cooperation consciousness, and reflection. The questionnaire dimension setting and topic determination are mainly based on “Learning and Study Strategies Inventory (LASSI)” and “College and University Classroom Environment Inventory (CUCEI)” (Opperman and Mason, 2020).

Then, the next step is to distribute the questionnaire, the students answer the questionnaire, and the head teacher cooperates to collect the questionnaire in a certain order. The questionnaire is classified and coded according to the students' academic performance. The head teacher takes the questionnaire and classifies the students into three categories: excellent, medium, and backward according to their academic achievements. The classification criterion is to divide the students' scores in the recent big exams into three categories: excellent, medium, and backward, and then code them. Excellent is 1, medium is 2, and backward is 3.

Likert's 5-point scoring method is used in the questionnaire, and the options are “very agree,” “agree,” “general,” “disagree,” and “very disagree,” which are set as 1, 2, 3, 4, and 5, respectively (Shin et al., 2018). For the teaching satisfaction questionnaire, in this exploration, the “robot experiment teaching satisfaction questionnaire” is designed and compiled from the five dimensions of learning resources, learning activities, communication and communication methods, learning results, and recognition of STEAM teaching. Table 2 shows some data results.

**Table 2:** Reliability statistics of the questionnaire.

	**Number of items**	**Sample size**	**Questionnaire response rate**
Q	19	41	100%
H	19	41	100%
A	24	39	95.12%
L	24	40	97.56%
P	24	41	100%

## Results and Analysis

### Questionnaire Test

In order to ensure the reliability and validity of the “comparison questionnaire” and “teaching satisfaction questionnaire,” two questionnaires are distributed to 41 students before formal teaching. In order to test the construct validity of the scale, factor analysis is needed. The purpose of factor analysis is to find out the potential structure of the scale, reduce the number of questions, and turn it into a group of fewer variables with greater correlation. As shown in Table 3, the Kaiser-Meyer-Olkin (KMO) index values of the “comparison questionnaire” and “teaching satisfaction questionnaire” are 0.695 and 0.701, respectively. According to Kaiser's point of view, when the KMO index value is above 0.6, factor analysis can be carried out; the Bartlett's test chi square value of the two questionnaires reaches a 0.05 level of significance, which proves that the research content is suitable for factor analysis. The above two aspects indicate that the data measured by the “comparative questionnaire” and “teaching satisfaction questionnaire” are suitable for factor analysis.

**Table 3:** KMO and Bartlett's test.

	**Sig**	**df**	**Approximate chi-square**	**Measure**
Comparison questionnaire	0.00	0.171	334.067	0.695
Teaching Satisfaction Questionnaire	0.00	0.276	693.217	0.701

Two questionnaires were compiled based on the study of much relevant literature, combined with actual research needs, teaching conditions, and other related content. After the completion of the initial questionnaire, the content validity of the two questionnaires is ensured by appropriate modification through the analysis of pre-survey items. The construct validity of the two questionnaires is discussed by the exploratory factor analysis method. Finally, it is concluded that the two questionnaires are suitable for factor analysis, and five factors are extracted from each questionnaire so that the excellent construct validity of the two questionnaires can be ensured.

**Figure 5:**
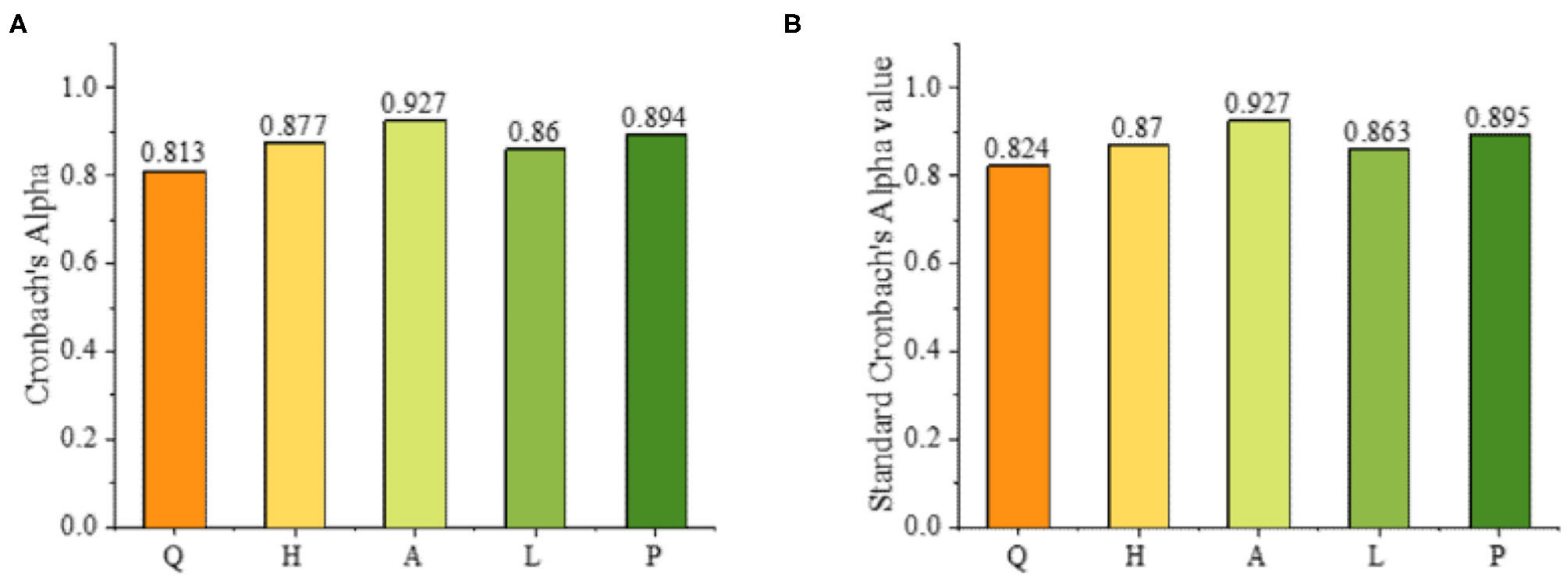
Reliability test results of questionnaire.

After the validity of the questionnaire is ensured, it is also necessary to test the reliability of the questionnaire. Cronbach's Alpha is used to test the internal consistency reliability of the scale. Figures 5A,B shows that the results of the questionnaires used have high stability and consistency.

### Comprehensive Quality Comparison Results

According to Table 4, from the overall perspective of pre-test and post-test, students have greater confidence in their own learning skills before the course (X = 8.2683) and after (X = 10.7073), followed by a strong interest in learning.

**Table 4:** Analysis of the differences before and after teaching.

	**Before teaching**	**After teaching**	***T***	***P***
Q	8.1951	10.3659	−5.653	0.00
H	8.2683	10.7073	−3.172	0.00
A	4.8293	6.9656	−6.354	0.00
L	5.1463	5.7805	−2.685	0.011
P	4.8049	6.6341	−7.649	0.00

### Teaching Satisfaction Results

**Figure 6:**
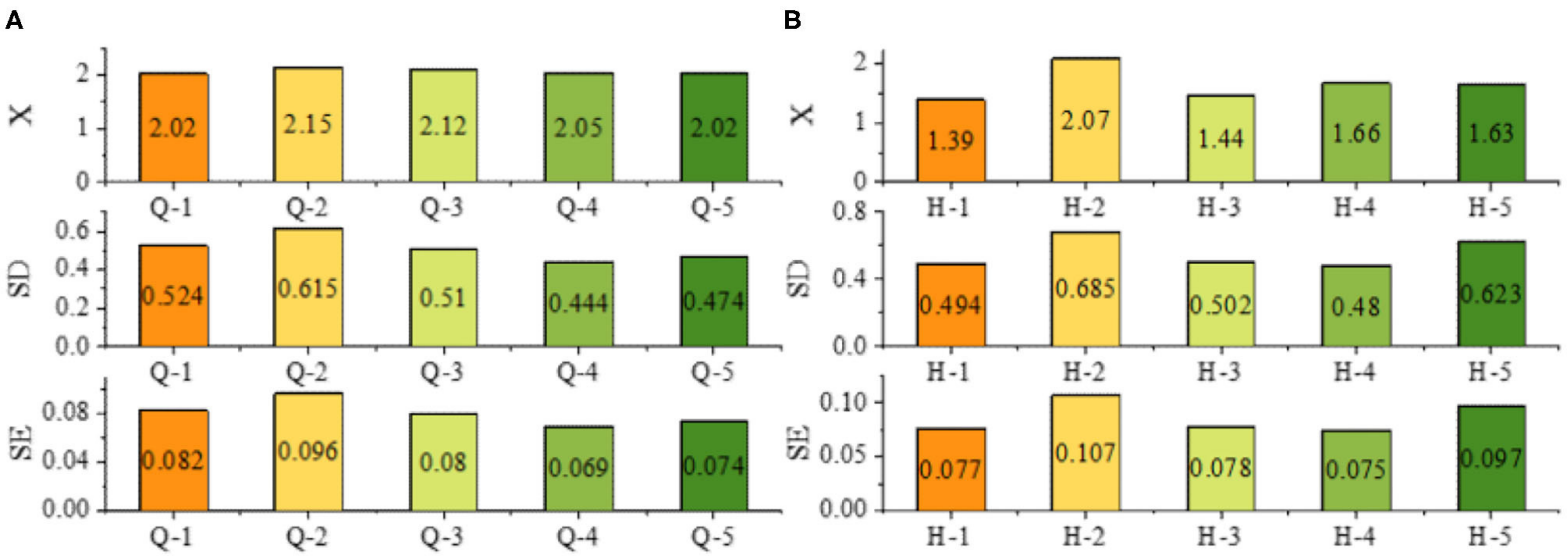
Learning interest and learning skill satisfaction.

The results in Figures 6A,B show that the students have a strong interest in learning the course before and after the course, and the learning interest is further strengthened after the course learning. There is a significant difference in the learning interest of the students in the robot experiment course (*P* = 0.000 < 0.05), and the learning interest after the course is higher than that before the course (*T* = 5.653 > 0). The results show that the learning interest of the students after the course is higher than that before the course and is more obvious. The students have a certain confidence in their own learning skills before the course, and the confidence is further strengthened after the end of the course; there are significant differences in the learning skills of the students before and after the course learning (*P* = 0.000 < 0.05), and the learning skills after the course are significantly improved compared with those before the course (*T* = 6.172 > 0). The results show that the students have mastered the corresponding learning skills in the learning process of the robot experiment course.

**Figure 7:**
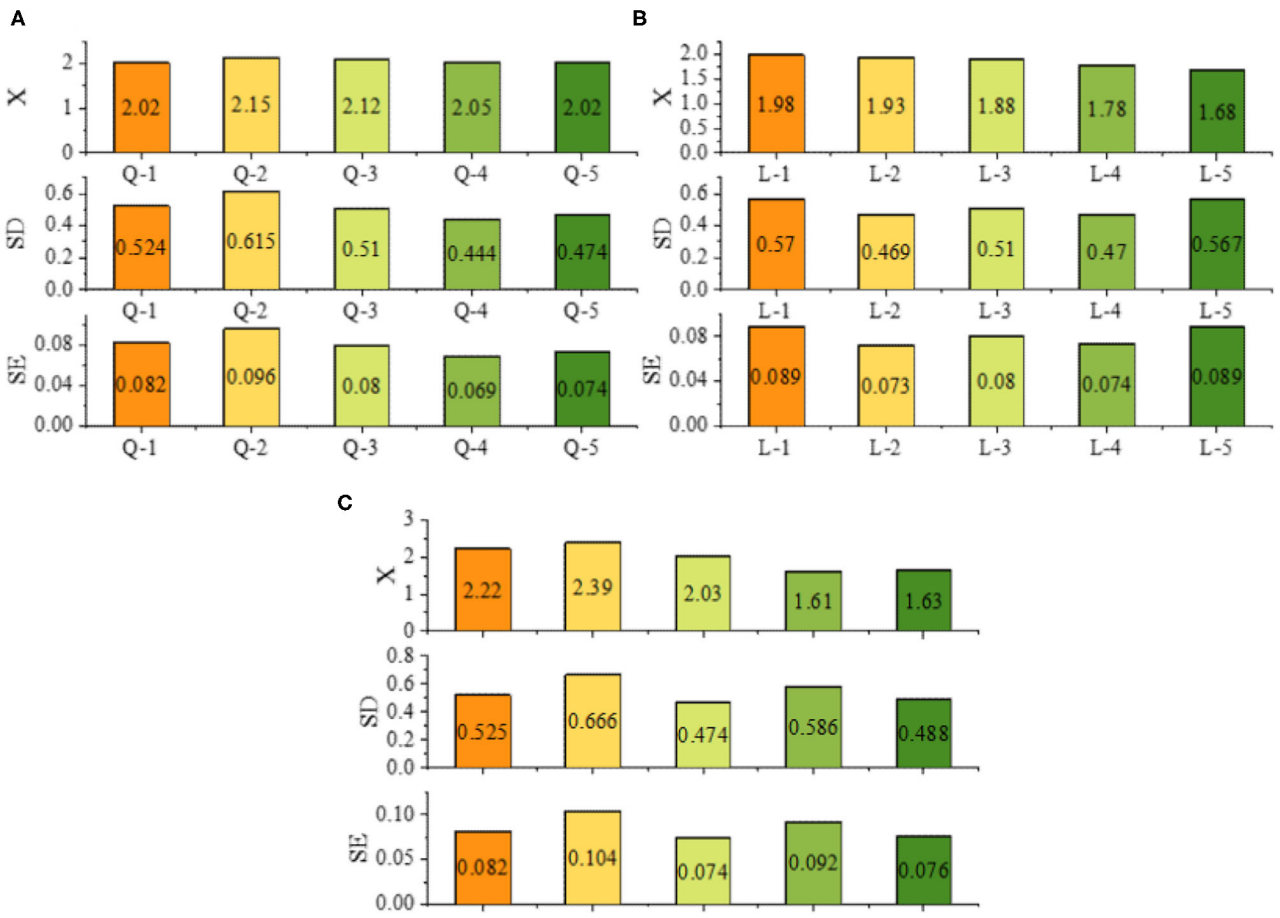
The results of cooperation consciousness, classroom participation, and satisfaction of after class style.

Figures 7A–C shows that most of the students are full of expectations for the teaching activities arranged by the teachers before the course. They think that they will actively participate in classroom activities. Moreover, the statistical results of the post-test data further prove this result: the average value of the post-test is lower than the corresponding average value before teaching, indicating that students are actively involved in the teaching and learning activities arranged for them by the teaching team. Most of the students have a strong sense of cooperation before the course; after learning the course, they have a stronger sense of cooperation. There is a significant difference in the awareness of cooperation before and after the course, and the awareness of cooperation has improved after the course. The results show that the cooperative consciousness of the students is improved by the learning robot experiment, but the range is small. When they encounter difficulties, problems and so on, they usually review, summarize, and reflect. These abilities gradually improve in the process of their course learning and group learning, and the growth rate rises.

### Analysis Results of Teaching Achievements

Table 5 shows that after the optimization of learning strategies, the learning performance of the experimental class is better than that of the control class, and the difference reaches a significant level (*t* = 2.243, df = 537, *p* < 0.05).

**Table 5:** Difference test results of the examination results in experimental class and the control class before and after the optimization of learning strategies.

**Teaching methods**	**Class type**	***N***	**Mean**	***t***	**df**	***P***
Before the optimization of learning strategies	Experimental class	54	619.110	0.420	536	0.675
	Control class	484	615.580			
After the optimization of learning strategies	Experimental class	54	594.780	2.243*	537	0.025
	Control class	485	574.230			

* *p < 0.05*.

Table 6 shows that, through the optimization of learning strategies, the learning performance of the experimental class has been significantly improved, with the average score rising to the first of 10 parallel classes.

**Table 6:** Examination results of experimental class and control class before and after learning strategy optimization.

**Class type**		**Before the optimization of learning strategies**	**After the optimization of learning strategies**
Experimental class	Mean	619.11	594.78
	*N*	54	54
	SD	73.71	74.66
Control class 1	Mean	608.65	581.17
	*N*	54	54
	SD	41.72	47.85
Control class 2	Mean	627.91	589.07
	*N*	54	54
	SD	46.48	49.46
Control class 3	Mean	626.48	575
	*N*	54	54
	SD	55.98	62.89
Control class 4	Mean	621.98	574.54
	*N*	54	54
	SD	49.60	58.38
Control class 5	Mean	622.85	571.06
	*N*	53	54
	SD	45.37	63.97
Control class 6	Mean	628.17	583.83
	*N*	54	54
	SD	40.79	49.36
Control class 7	Mean	614.62	577.49
	*N*	53	53
	SD	37.49	36.53
Control class 8	Mean	588.85	553.52
	*N*	54	54
	SD	98.70	105.01
Control class 9	Mean	600.87	562.46
	*N*	54	54
	SD	58.29	61.75
Total	Mean	615.94	576.29
	*N*	538	539
	SD	58.50	64.10

Table 7 shows the experimental class's population distribution structure and the control class at these three grades. According to the above chart, the number of excellent students decreases, and the number of backward students increases in the control class; the number of excellent students increases while the number of backward students decreases in the experimental class. It shows that after the optimization of learning strategies, the overall academic achievement of the experimental class students has improved. This shows that the training mode of international service design talents based on an HCI intelligent robot from the perspective of cognitive psychology is more successful.

**Table 7:** Grade distribution of students' academic achievement in experimental class.

	**Before the optimization of learning strategies**		**After the optimization of learning strategies**	
**Grade**	**Students number**	**Proportion (%)**	**Students number**	**Proportion (%)**
Excellent	14	25.9	18	33.3
Medium	27	50.0	27	50.0
Backward	13	24.1	9	16.7

## Discussion

Through the suggestions and problems put forward by the research objects, it is found that the lack of teaching resources, the shortage of teachers, and the inadequate consideration of the details in teaching design and teaching methods will create certain losses in terms of the learning experience, learning process, and even learning results. Therefore, the teaching team must pay enough attention to this point and fully grasp all aspects of teaching design in future teaching and learning activities. Of course, the existence of problems is the best motivation for self-improvement, and the suggestions put forward by students are the most valuable wealth for the improvement of teaching. While the learners are required to grow up, educators also need to continually develop to ultimately achieve the common growth of both teachers and students. The expectation expressed by the learners is the greatest affirmation to the teaching team. They have learned something during the experimental course and expect to have more opportunities to learn in the future. This shows that the teaching team's contribution is valuable, and the teaching team is also full of a sense of achievement so that the teaching team has a greater passion for continuing to improve the teaching design and students can acquire more knowledge and skills.

The four aspects can be summarized as three points. First, the research objects give a positive evaluation of the whole teaching and learning activities. In the reflection diary, learners clearly describe the teaching content and experimental tasks, actively participate in teaching and learning activities, and recognize the teaching design and teaching implementation of the teaching team. Second, the object of study has gained and learned something. During the course, learners not only enrich the knowledge system and cognitive structure but also get inspiration from daily life and cultivate their own innovative thinking. At the same time, it has cultivated the creative consciousness, improved practical ability, and enhanced the sense of teamwork. Third, in view of the shortcomings in the teaching process, some constructive suggestions are put forward. These suggestions can urge the teaching team to design teaching more carefully, provide high-quality teaching resources, and constantly optimize teaching and learning methods so as to promote meaningful learning of learners.

## Conclusion

The talent training mode of international service design has been studied. The background of the development of educational information technology in China has been understood. Through the study of relevant policy documents, major events, and documents, the revolutionary impact of information technology on educational reform has been analyzed. It is found that, although the use of the domestic educational robot starts relatively early, most educators are more focused on teaching the course than using the robot. Relatively speaking, there have been few studies made on creating a language learning environment and assisting language teaching by using robots, which, to a certain extent, lacks theoretical guidance and empirical research. Therefore, the robot-aided design talent training mode is taken as the research object, trying to bring a new scheme with research data support for the studies into robot-aided teaching, which is also the innovation point of this study. First, the research background and significance are described, and the current development of robot education and service design is introduced. Then, the related concepts, such as AI, robot, talent training mode, and cognitive psychology, are analyzed, and the application of AI, the robot, and cognitive psychology in talent training is analyzed theoretically. Finally, the talent training mode of international service design by AI technology and robot is studied, and the talent training mode of service design with the robot as auxiliary education way is established. Through the survey, the application of a robot in the talent training mode of international service design is analyzed. The results show that robot-assisted talent training is a feasible way to improve the efficiency of talent training.

Due to the limited knowledge level, there is a lack of comprehensiveness and depth in the research process. The focus is to analyze the application of cognitive psychology, AI, and other related theories and technologies in talent training, which lack specific research on AI technology and human–computer interaction technology. It is hoped that in the follow-up research, the AI technology and human–computer interaction technology can be specifically analyzed so as to improve the current technology deficiencies. This study lacks practical application of a human–computer interaction-based intelligent service robot in the process of talent training, and it is more inclined to theoretical analysis. It is hoped that, in the follow-up research process, the talent training mode can be applied in practice to observe the specific application effect of the designed talent training mode and improve the deficiencies of this study.

## Data Availability Statement

The raw data supporting the conclusions of this article will be made available by the authors, without undue reservation.

## Ethics Statement

The studies involving human participants were reviewed and approved by Taiyuan University of Technology Ethics Committee. The patients/participants provided their written informed consent to participate in this study. Written informed consent was obtained from the individual(s) for the publication of any potentially identifiable images or data included in this article.

## Author Contributions

The author confirms being the sole contributor of this work and has approved it for publication.

## Conflict of Interest

The author declares that the research was conducted in the absence of any commercial or financial relationships that could be construed as a potential conflict of interest.
